# Hyponatremia: A Tip of the Iceberg in the Late Diagnosis of Tuberous Sclerosis Associated With Lymphangioleiomyomatosis

**DOI:** 10.7759/cureus.20131

**Published:** 2021-12-03

**Authors:** David Paiva, Anabela De Carvalho, Ana Luísa Campos, Filipa Gonçalves, Cristina Silva, Olinda Miranda, Sandra Barbosa, Jorge Cotter

**Affiliations:** 1 Internal Medicine, Hospital da Senhora da Oliveira Guimarães, Guimarães, PRT

**Keywords:** lymphangioleiomyomatosis, polydipsia, angiomyolipomas, hyponatremia, tuberous sclerosis

## Abstract

Tuberous sclerosis (TS) is a rare, autosomal dominant, multisystem genetic disease that causes multiple benign tumors in the brain and other vital organs. Rarely, it can be associated with lymphangioleiomyomatosis (LMA) that is characterized by the proliferation of immature smooth muscle cells in the walls of the airways, venules, and lymphatic vessels in the lung. Here, we present the case of a 44-year-old intellectually disabled woman with a history of marked polydipsia who presented to the emergency department with persistent vomiting. She was hemodynamically stable and did not have any fever. The analytical study showed severe and symptomatic hyponatremia. On physical examination, multiple skin lesions compatible with angiofibromas were noted and the diagnosis of TS was made (confirmed with the genetic study). The multiorgan study documented the presence of multiple cystic images in the lung parenchyma associated with LMA. The aim of this case report is to highlight the importance of targeting cutaneous lesions for a rapid diagnosis of this pathology and to identify the etiology of a severe (symptomatic) ionic disorder and referral to a multidisciplinary team.

## Introduction

Tuberous sclerosis (TS) (also known as tuberous sclerosis complex) is a rare, autosomal dominant, multisystem genetic disease that is characterized by non-cancerous (benign) tumors that grow in the brain and other vital organs such as the kidneys, heart, eyes, lungs, and skin [1]. In rare cases, it is associated with lymphangioleiomyomatosis (LMA), which is characterized as a multisystem, autosomal dominant pathology that essentially affects females. LMA is caused by the abnormal growth of smooth muscle cells, especially in the lungs, pleurae, and lymphatic system, leading to distortion of the lung architecture, cystic emphysema, and progressive deterioration of lung function as well as other extrapulmonary manifestations (angiomyolipomas and lymphatic tumors) [2].

The aim of this article is to describe a rare case of a woman with a cognitive disability and atypical clinical manifestations whose diagnosis was based on the lesions identified during a physical examination that had not been evaluated until then.

## Case presentation

A 44-year-old woman with intellectual disability and a history of multiple seizures during childhood presented to the emergency department with persistent vomiting. She denied the presence of pain, fever, or a family history of similar symptoms. There were no documented seizure episodes.

**Figure 1:**
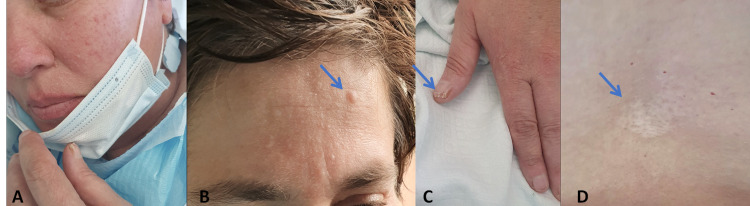
Physical examination findings. (A and B) Angiofibromas on the face. (C) Hamartomas on hand nails. (D) Shegreen patch-like lesion.

On examination, she was hemodynamically stable and did not have any fever. Pulmonary and cardiac auscultation as well as the abdomen had no alterations on physical examination. Multiple skin lesions on the face were documented, compatible with angiofibroma. In addition, hypopigmented lesions were documented on the legs and lumbar region, compatible with ash leaf spot, Shegrenn patch-like lesions were noted on the back, and hamartomas were observed on hand nails (Figure 1).

The analytical study showed severe hyponatremia at 107 mEq/L (normal: 135-145 mEq/L), hypokalemia at 2.81 mEq/L (normal: 3.5-5.1 mEq/L), and hypochloremia at 68 mEq/L (normal: 95-105 mEq/L). Plasma osmolarity was 230 mOsm/L with urinary osmolarity of <70 mOsmol/kg and urinary sodium of 23 mEq/L, compatible with euvolemic hypoosmolar hyponatremia. The patient was admitted for surveillance and etiologic investigation.

In the presence of euvolemic hypoosmolar hyponatremia and low urinary osmolality, the main diagnostic hypotheses included primary polydipsia, malnutrition with low intake of dietary solutes (e.g., beer potomania, extreme vegetarian diets), and reset osmostat variant of inappropriate antidiuretic hormone secretion. After careful history taking with the patient’s mother, excessive water consumption was reported. No pharmacological causes were found. Polyuria was observed during hospitalization (output greater than 4 L/min). Fluid suppression was performed, and after eight hours urinary osmolarity was measured again with correction for values >300 mOsm/L. Hence, the diagnosis of primary polydipsia was assumed.

**Figure 2:**
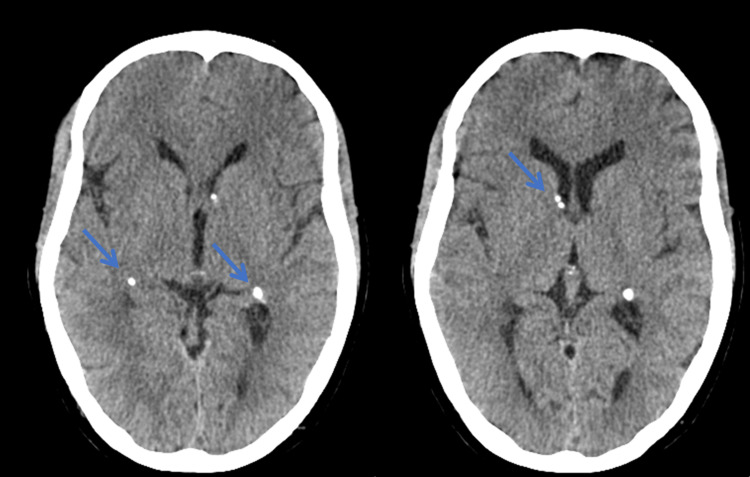
Cranioencephalic CT. Subependymal, non-specific gross calcifications along the walls of the lateral ventricles and predominant right parietal subcortical hypodensities (blue arrow). CT: computed tomography

Cranioencephalic computed tomography (CT) was performed which demonstrated the presence of subependymal, non-specific gross calcifications along the walls of the lateral ventricles and predominant right parietal subcortical hypodensities (Figure 2).

**Figure 3:**
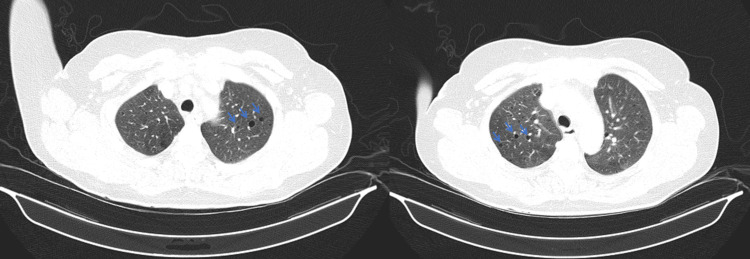
Thoracic CT. Multiple cystic images in the lung parenchyma (blue arrow). CT: computed tomography

**Figure 4:**
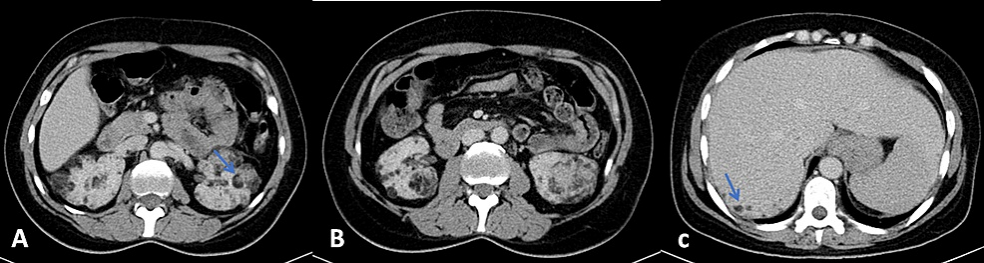
Abdominopelvic CT. Multiple nodules in the parenchyma of both kidneys compatible with angiomyolipomas (A and B, blue arrow). In the liver parenchyma, some nodular formations (C, blue arrow) corresponding to intrahepatic angiomyolipomas can be seen. CT: computed tomography

Thoracoabdominopelvic CT to study multiorgan involvement confirmed the presence of aspects suggestive of TS manifestations, namely, multiple cystic images in the lung parenchyma, probably related to LMA phenomena, without other relevant parenchymal changes (Figure 3). The kidneys demonstrated multiple nodular images with negative density, suggestive of angiomyolipomas; however, other etiologies could not be ruled out (Figure 4). Echocardiogram had no significant alterations.

Due to the documented renal alterations and taking into account the higher risk of renal cell neoplasm in patients with TS, abdominopelvic magnetic resonance imaging (MRI) was performed to better characterize the cystic formations. In the liver parenchyma, some nodular formations (the most prominent approximately 10 mm in diameter in segment VII of the right hepatic lobe, hypointense on T1 (phase opposition)) were documented, which was thought to correspond to intrahepatic angiomyolipomas. Multiple nodules were found in the parenchyma of both kidneys, consisting of adipose tissue, compatible with angiomyolipomas. On the anterior aspect of the middle third of the left kidney, two more nodular formations, practically contiguous, approximately 2.7 cm and 3 cm in diameter, were identified, along with another nodule in the inferior pole measuring 6 cm, which was partially exophytic and deforming the renal contour. These lesions were partially constituted by adipose tissue, suggesting that they were more likely to correspond to angiomyolipomas.

To complete the etiological study, a genetic study was performed to search for mutations in the *tuberous sclerosis complex* (*TSC*) 1 and 2 genes, which was positive (the c976-15G>A variant of the *TSC2* gene was detected). Therefore, a diagnosis of LMA was confirmed in the context of TS during hospitalization.

The patient was observed by psychiatry, and she was started on valproic acid to control behavior. We explained to the patient and her mother the importance of water restriction up to 1.5 L/day. She was discharged to Internal Medicine and Psychiatry consultation for a continuation of her clinical guidance. At the first reassessment, the patient had normalized sodium levels (142 mEq/L), with no new complications.

## Discussion

TS is a rare, autosomal dominant, multisystem disease characterized by seizures (present in approximately 80-90% of patients), cognitive delay (autism, behavioral, and psychiatric disorders), and brain, heart, skin, and kidney tumors. The diagnosis can be made clinically or by genetic testing. This disease is caused by defects, or mutations, on two genes, namely, *TSC1* and *TSC2*, which code for the proteins hamartin and tuberin, respectively. Skin involvement is crucial for suspecting the diagnosis of TS. Most patients are diagnosed due to the presence of neurocutaneous stigmata or seizures [1,3,4].

Rarely, in one-third of TS cases, it can be associated with LMA, affecting almost exclusively females. Its pathology is characterized by the proliferation of immature smooth muscle cells in the walls of airways, venules, and lymphatic vessels in the lung. The estimated incidence of LAM is between 1 and 2.6 cases per 1,000,000 women. The two most common symptoms of LAM are dyspnea or pneumothtorax, but other symptoms can occur (e.g., hemoptysis, non-productive cough, chylous pleural effusion, and chylous ascites). The diagnosis of LMA is made by a high-resolution CT scan, and tissue confirmation is not necessary [2].

This case describes a patient with cognitive delay and behavioral disorder characterized by marked polydipsia who was admitted due to a severe electrolyte disorder (symptomatic hyponatremia), interpreted in the behavioral context. Despite episodes of epilepsy reported during childhood, there were no new episodes in adulthood. Physical examination and documentation of skin changes raised the initial suspicion of TS, which was confirmed with the genetic study (although not mandatory for the diagnosis). A multisystemic study verified the presence of multiple angiomyolipomas, namely, in the lungs, liver, and kidney, which were highly suggestive of LMA. Despite being an asymptomatic patient, diagnosed at 44 years of age, her follow-up in consultation needs to be maintained to monitor for worsening pulmonary involvement and to prevent associated complications.

## Conclusions

This case is interesting not only because of the late diagnosis but due to the suspicion during the etiological study of an electrolyte disorder, which fits into the cognitive disorder that is characteristic of this pathology. TS-LMA is a rare but potentially debilitating disease; hence, it is crucial to alert physicians about the cutaneous characteristics to allow an early diagnosis, as well as referral to multidisciplinary care to prevent complications, reduce morbidity, and study the multiorgan involvement.
